# Vitamin D supplementation and hemoglobin: dosing matters in prevention/treatment of anemia

**DOI:** 10.1186/s12937-021-00680-x

**Published:** 2021-03-19

**Authors:** Lena M. Napolitano

**Affiliations:** University of Michigan Health System, Room 1C340-UH, University Hospital, 1500 East Medical Drive, SPC 5033, Ann Arbor, MI 48109-5033 USA

*To the Editor:*

Arabi et al. report in the recent systematic review and meta-analysis of the effect of vitamin D supplementation on hemoglobin concentration that supplementation with vitamin D had no significant effect on hemoglobin and ferritin levels, while positive effects on transferrin saturation and iron status were observed [1]. There is concern that the studies in this meta-analysis included highly variable patient cohorts and vitamin D dosing, acute and chronic treatment strategies, and not all patients had anemia.

The potential beneficial impact of vitamin D on hemoglobin in patients with anemia was not separately examined. Vitamin D supplementation may provide a safe, simple and cost-effective therapy for prevention and/or treatment of anemia. There is concern that the conclusion of this meta-analysis “… vitamin D had no significant effect on hemoglobin levels” is not meaningful based on the wide diversity of studies included. Why would vitamin D impact on hemoglobin if hemoglobin levels are normal?

Did the authors consider assessment of the total vitamin D dose in each of the studies, and separate analysis of the trials in anemic patients? The Table below confirms the wide variability in total dose and duration in these randomized clinical trials, which makes it challenging to discern an impact of vitamin D on hemoglobin in anemic patients.

It has clearly been documented that *high-dose* vitamin D is required for hepcidin suppression, which is required for optimal increase in endogenous erythropoiesis and subsequent increase in hemoglobin in anemic patients who have high hepcidin levels and anemia of inflammation or chronic kidney disease [2–7]. We would like to highlight the very important finding in Fig. 2 (effect of vitamin D on hemoglobin) in 2.3.6 “Critically ill patients”, that the placebo cohort mean hemoglobin was 6.7 g/dL vs. 11.25 g/dL in the high-dose (500,000 units total, 100,000 units daily for 5 days) vitamin D intervention cohort.

It should also be noted that in Table 1 for the Smith study in mechanically ventilated critically ill adults, the duration of the vitamin D intervention was listed as “4 weeks”, but the study drug was administered in 5 equal doses over 5 days, not 4 weeks. There are additional errors in that Table regarding drug dosing, duration and year of publication, all corrected in the Table below.

**Table 1:** Wide variability in Vitamin D dose and duration, type of patients enrolled, and whether anemia was present or not in randomized clinical trials

	Patients	Anemia	Vitamin D dose	Vitamin D duration	Total Vitamin D dose
Trautveffer 2014	Healthy adults	No	Fortified bread with 400 IU vit D3 daily	8 weeks	22,400 IU vit D3
Miskulin 2016^a^	Adults with CKD	Yes	Oral dose of 50,000 IU vit D2 weekly	6 months	1,200,000 IU vit D2
Toxqui 2013	Adult Women	Yes	Fortified milk with 200 IU vit D3 & 15 mg iron daily	16 weeks	22,400 IU vit D3
Sooragonda 2015	Healthy adults	Yes	0.6 Lakh Amp vit D3+ Amp iron once	Once	600,000 IU vit D3
Smith 2017 [7]^a^	Healthy adults	No	Oral dose of 250,000 IU, vit D3 once	Once	250,000 IU vit D3
Smith 2018 [6]	Critically ill adults	Yes	Enteral dose of 50,000 IU (I1) or 100,000 IU (I2) vit D3 daily	5 days	500,000 IU (I1) vit D3 250,000 IU (I2) vit D3
Madar 2016	Healthy adults	No	I1 = tablet 1000 IU, vit D3 daily I2 = tablet 400 IU, vit D3 daily	16 weeks	42,000 IU (I1) vit D3 16,800 IU (I2) vit D3
Hennigar 2016	Healthy adults	No	Fortified bar with 1000 IU vit D3 + 2000 mg calcium, 2 bars per day	9 weeks	126,000 IU vit D3
Ernest 2016	Adults with hypertension	No	Oral dose of 2800 IU vit D3 daily	8 weeks	156,800 IU vit D3
Ernest 2017	Adults with heart failure	Some (23 of 172)	Oral dose of 4000 IU vit D3 daily	36 months	4,032,000 IU vit D3
Jastrzebska 2017	Healthy adults	No	Oral dose of 5000 IU vit D3 daily	8 weeks	280,000 IU vit D3
Dahlquist 2017	Healthy adults	No	Sport drink with 5000 IU vit D3 once	Once	5000 IU vit D3
Walentukiewicz 2018^a^	Elderly women	No	Oral dose of 28,000 IU vit D3 weekly	12 weeks	336,000 IU vit D3
Panwar 2018	Adults with CKD	Yes	Oral dose of 0.5 mcg (20 IU vit D3) calcitriol daily	6 weeks	840 IU vit D3

*CKD* Chronic kidney disease

^a^Hemoglobin not reported

## Data Availability

Not applicable.
